# Internal Jugular Vein Phlebectasia Presenting with Hoarseness of Voice

**DOI:** 10.1155/2013/386961

**Published:** 2013-11-28

**Authors:** Sohini Chakraborty, Pranab Kumar Dey, Amrita Roy, Nilay Ranjan Bagchi, Debalina Sarkar, Sumita Pal

**Affiliations:** Department of Paediatrics, Medical College, Kolkata, India

## Abstract

Internal jugular phlebectasia presents as a soft cystic mass in the neck that appears on straining. We present a case of a 7-year-old girl who presented with a painless soft cystic mass in the neck associated with hoarseness of voice. Based on clinical examination and CT image, diagnosis of right internal jugular phlebectasia was made.

## 1. Introduction

Jugular vein phlebectasia (JVP) also known as venous congenital cyst, venous aneurysm, venous ectasia, or essential venous dilatation refers to a congenital fusiform or saccular dilatation of the jugular vein that appears as a soft, compressible mass in the neck on straining like coughing, crying, and sneezing or may be triggered by the Valsalva maneuver. The other possibilities of such a swelling include laryngocele, branchial cyst, cystic hygroma, cavernous hemangioma, and superior mediastinal mass [1]. Jugular vein phlebectasia usually presents on the right side, and most patients are children, boys being twice as more often affected as girls [2]. It is a benign condition and is usually asymptomatic [2]. The possible causes of JVP are gross anatomic abnormality, mechanical compression or trauma of vein, congenital structural defects, and idiopathic [3]. Phlebectasia has been reported in all neck veins internal jugular, external jugular, anterior jugular, and superficial communicants in order of decreasing frequency [2]. Absence of a wide mediastinum or air in the mass on simple chest films eliminates mediastinal tumor or laryngocele, respectively. Noninvasive methods of diagnosis include ultrasonography combined with Doppler flow imaging and spiral computerized tomography scan with contrast. Treatment is mainly conservative in cases where there are no symptoms or complications. Surgical treatment, usually done for aesthetic purposes, consists of excision of dilated portion of the vein or unilateral excision of the vein. Thrombosis and Horner's syndrome are the reported complications [4].

## 2. Case Report

**Figure 1:**
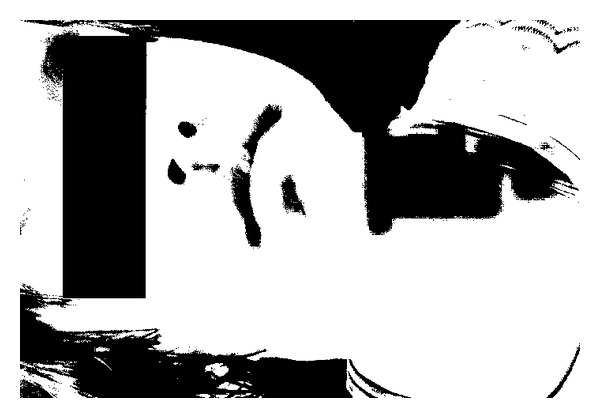
A 4 × 4 cm mass appeared on her right side of the neck on straining (Valsalva maneuver).

**Figure 2:**
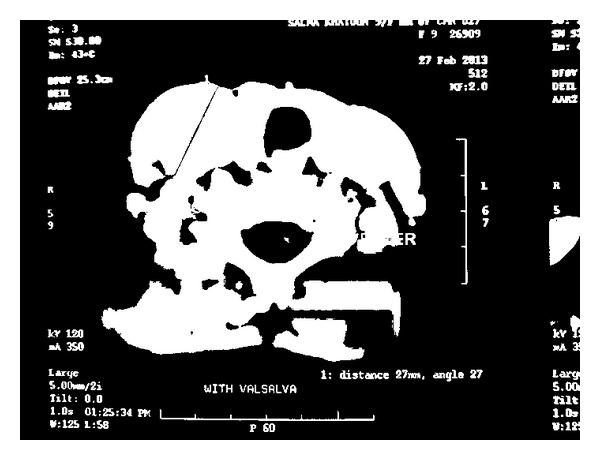
A contrast enhanced CT scan showing ectasia of the right internal jugular vein.

A 7-year-old girl presented to us with complaints of a painless swelling appearing in the neck on coughing or sneezing and disappearing at rest since last 1 year. It was gradual in onset and slowly progressive in nature. It was associated with hoarseness of voice for the same duration. However, there was no history of pain, fever, facial puffiness, difficulty in breathing, or swallowing. She had no history of trauma to the neck region or any previous neck infection. On physical examination, the child looked healthy. Otologic, rhinoscopic, and oropharyngeal examination revealed no abnormality. On initial examination of neck, no identifiable mass was seen. A 4 × 4 cm mass appeared on her right side of the neck on straining (Valsalva maneuver); the mass emerged from below the right sternocleidomastoid muscle and extended up to the right anterior triangle of the neck (Figure 1). The swelling was soft, cystic, and nontender; skin over nodes was normal. No bruit or pulsation over the swelling was present. The mass was not transilluminant. It was not possible to get below the swelling. Other systemic examinations were unremarkable. Chest X-ray did not show any mediastinal widening or any air in the region of the mass. Laryngoscopy was done which revealed no abnormality. A contrast enhanced CT scan (Figure 2) was done which confirmed the diagnosis of ectasia of the right internal jugular vein. The parents were reassured and no surgical treatment was advised. Voice therapy was advised, leading to symptomatic improvement.

## 3. Discussion

Venous ectasia in the neck is a rare entity, especially in children [2]. Internal jugular ectasia was first described by Zukschwerdt and subsequently characterised by Gerwgi. The term phlebectasia indicates abnormal outward dilatation of the vein without tortuosity and differs from varix which implies both dilatation and tortuosity. The internal and external jugular veins are generally affected. However, there are reports of anterior jugular vein ectasia. In our case the internal jugular vein was affected which is more common [2]. It is reported that males are more commonly affected than females [2]. Our case was of a 7-year-old girl. LaMonte et al. [1] hypothesized that the ectasia is more common on the right side because the right innominate vein lies in contact with the right apical pleura. Hence, any increase in intrathoracic pressure could be directly communicated to the right IJV. The left being placed more medially was not subject to this stress. Our patient presented with right sided swelling.

Clinically, the mass appears as a soft, cystic, and fusiform mass that appears on straining and completely disappears at rest. In our case it was associated with hoarseness of voice. There are very few case reports reporting this complication [5]. There are mainly three types of swelling which distend on Valsalva and disappear completely at rest: (a) tumors or cysts of the superior mediastinum, (b) external laryngeal diverticulum and laryngocele, and (c) venous enlargement of the superior vena caval system [6]. Laryngocele is most common among these. In our case absence of mediastinal widening or air in the mass excludes mediastinal tumor and laryngocele. Laryngoscopy was done which revealed no abnormality. Coloured Doppler ultrasonography is sufficient to confirm phlebectasia [6], computerized tomography may also be used as done in our case. Usually histopathologic studies are normal. Sometimes disordered arrangement in smooth muscle cells, elastic tissue, and connective tissue may be seen [7].

The swelling is not known to progress rapidly and there have been no instances of spontaneous rupture of the swelling [1, 8]. Complications reported are thrombosis and Horner's syndrome [4]. Balik et al. [9] reported a case that had jugular phlebectasia with thrombosis and suggested surgical removal of the involved segment without delay because of thrombosis and some other unknown potential complications. Our case had no such complications.

Unless complications occur or the lesions are cosmetically deforming, most authors recommend conservative management [1, 10]. Surgery is indicated for cosmetic reasons and in symptomatic patients only [2]. Surgical procedures include ligation of the affected vein which is the standard procedure and there is no unwanted sequelae. As our patient was asymptomatic, we counselled the parents and reassured them of good prognosis and no further steps were taken. Voice therapy was advised for symptomatic management.

Neck masses are commonly seen in children, and though phlebectasias are relatively uncommon, this benign condition must be kept in mind to avoid unnecessary investigations and risky surgical procedures.
